# Prevalence of cytotoxin-associated genes of *Helicobacter pylori* among Iranian GERD patients 

**Published:** 2017

**Authors:** Aref Shavalipour, Habib Malekpour, Hossein Dabiri, Hossein Kazemian, Homayon Zojaji, Mahboube Bahroudi

**Affiliations:** 1 *Department of Medical Microbiology, Shahid Beheshti University of Medical Sciences, Tehran, Iran*; 2 *Basic and Molecular Epidemiology of Gastrointestinal Disorders Research Center, Research Institute for Gastroenterology and Liver Diseases Shahid Beheshti University Medical Sciences, Tehran, Iran*; 3 *Department of Medical Microbiology, School of Medicine, Tehran University of Medical Sciences, Tehran, Iran *; 4 *Gastroenterology and Liver Diseases Research Center, Research Institute for Gastroenterology and Liver Diseases, Shahid Beheshti University of Medical Sciences, Tehran, Iran*

**Keywords:** *Cag A*, *Cag E*, *Helicobacter pylori*, GERD, Iran

## Abstract

**Aim:**

Since the impact of *H. pylori* and its virulence is not clear in GERD, this study aimed to evaluate the prevalence of *cag A* and *cag E* gens of *H. pylori* among Iranian GERD patients.

**Background:**

Gastroesophageal reflux disease (GERD) is defined as a condition of reflux the stomach juice by low pH causes tissue damage. *Helicobacter pylori* may or may not influence the GERD; however, it is unclear.

**Methods:**

This study was a case-control study performed on patients with GERD who underwent upper gastrointestinal endoscopy at Taleghani Hospital of Tehran, Iran. Prevalence of H. pylori and presence of the cag* A* and *cag E* genes in GERD and control group was investigated.

**Results:**

*H. pylori* was detected in 54% and 62% of GERD and control groups respectively. Prevalence of *cag A* gene among GERD patients was 44.4% whereas among the control group it was 87%. Prevalence of the *cag E* among GERD patients and control group was 44.4% and 64% respectively. Coexistence of *cag A* and *cag E* in GERD patients was 25.7% and in the control patients it was 54.8%.

**Conclusion:**

We did not find correlation between *H. pylori* existence in GERD patients in comparison to the control group. Similar to other Asian studies, the presence of the c*ag A* in control group was more than GERD patients significantly. The co-existence of *cag A* and *cag E* was also more in control group significantly.

## Introduction

 Gastro esophageal reflux disease (GERD) is defined as a condition in which the reflux of the stomach juice by low pH causes tissue damage. GERD’s prevalence in Western countries is about 20% and it is 2.5% – 6.7% in Asian population; however, the prevalence is increasing in Asian populations (1). According to previous studies, *Helicobacter pylori* may or may not influence the GERD; however, it is unclear (2).

*H. pylori* is a Gram negative micro-aerophilic spiral shaped bacteria that colonize the gastric lumen of humans and other primates (3). Infection with *H. pylori* occurs worldwide, but the geographical prevalence varies greatly from 90% in developing countries to 20–50% in developed countries (4).

 It can be the major cause of peptic ulcer and gastritis and is known to have a relation with some infectious and non-infectious diseases, such as parasitic infection, malignancy, autoimmune thyroid disease, and GERD (4-6). 

Cytotoxin-associated gene products (*Cag A* and *Cag E*) are virulence factors of H.pylori which contribute to disease progress (7). Previous studies suspected that *Cag A* of H.pylori plays a role in the pattern of infections and diseases such as GERD (8, 9). Polymerase chain reaction (PCR) technique that has sensitivity and specificity for the diagnosis of *H.pylori* is also pathologic methods has these characteristics so these are the gold standard for diagnosis of *H.pylori* (37،^38^،^39^).

Many epidemiological studies demonstrated a negative association between *H. pylori* infection and GERD. Some of the virulence factors, such as *Cag A*, may affect the diverse prevalence of GERD. A high incidence of *Cag A*-positive isolates has been reported in Asian population. The variety of *H. pylori* infection in Eastern and Asian countries among GERD patients is attributed to *Cag A* gene. So far, many studies have evaluated the effects of *H. pylori* eradication among GERD patients, but it is inconclusive. Eradication of *Cag A *positive H*. pylori* leads to recovery of acid secretion capacity and corpus gastritis which might be the causes of the higher incidence of GERD in Asian population (2). Of the cag pathogenicity island genes, the *cagE* gene (cytotoxin associated gene E), is also related to an increased production of IL-8 in the gastric epithelial cells (41). Thereby the *cagE* is an important marker of pathogenicity alone or combined with *cagA* (40).Since the impact of *H. pylori* and its virulence in GERD development is not yet well clear, so we aimed to evaluate the prevalence of *H. pylori* as well as its major Cag pathogenesity island markers including *Cag A *and* Cag E* gens in GERD patients compare to control group referring to Taleghani Hospital, Tehran, Iran. 

## Methods

**Participants**

The current case-control study was performed on patients who had undergone endoscopy at Taleghani Hospital of Tehran, Iran during one year (2014).

**Table 1:** Primers sequences used in the current study

Gene	Primer designation	Sequence	Reference
*Ure C*	*Ure C*R1 *Ure C*F1	GCTTACTTTCTAACACTAACGCGC GGATAAGCTTTTAGGGGTGTTAGGGG	37
*Cag A*	*CagA* F1 *CagA* R1	AACAGGACAAGTAGCTAGCC TATTAATGCGTGTGTGGCTG	37
*Cag E*	CagE F1 CagE R1	GCGGCAGTAACCAACCTTATCA CAAGCCCATTAGGATCATTGTG	36

According to various studies in Iran, the average prevalence of GERD patients was 27% (10). The number of patients with GERD symptoms was calculated with the following formula: N= z^2^ P (1-P)/ d^2^. 

(Prevalence (P) = 0.27; z = 1.96; d = 0.05)

**Table 2:** Prevalence of the c*ag A* and *cag E* genes among GERD and control patients

Patients	number	*H. pylori*		Virulence genes (%)		
		*negative*	*positive*	*Cag A*	*Cag E*	Co-presence
GERD	50	23	27	12(44.4)	12(44.4)	7(25.7)
Control	50	19	31	27(87)	20(64)	17(54.8)
P value	-	0.54		0.0007	0.18	0.03

Therefore, during the study, 303 cases were investigated. All patients were examined by a gastroenterologist. Also questionnaires was loaded for each patient prior to biopsy. Based on gastroenterologist's diagnosis (upper gastrointestinal endoscopy and physician examination), only 50 patients were diagnosed with GERD. Of other patients without Gastro esophageal reflux disease, 50 patients were considered as control group equal to GERD group. The presence of *H. pylori* infection in the subjects was determined by histological examination and detection of the *ure C* gene by polymerase chain reaction (PCR).

**Biopsy**

After a fasting period, upper endoscopy was performed with a standard forward-viewing endoscope. After inspection of the entire gastric mucosa, multiple biopsies were taken from the stomach. During endoscopy samples taken from the gastric antrum were placed in the the sterile microtube and was transferred to the laboratory. The specimens were preserved at -20 C for next steps.

**Histological examination**

The biopsy samples of the gastric antrum destined for histology were fixed in formalin and stained with Hematoxylin-Eosin (H&E) and Giemsa. (42)

**DNA extraction and PCR performing**

DNA of biopsy samples was extracted by DNA extraction kit (DNeasy 96 Blood & Tissue Kit, Qiagen, USA). To confirm the presence of *H. pylori* among samples, PCR reaction was performed for *Ure C* gene. After identification of *H. pylori* positive samples, PCR was carried out for *cag A *and* cag E* genes as described previously (11) (Table 1 the primer were mentioned) (36).

**Statistical analysis**

All data were analyzed using SPSS 22. For compression of the presence of *H. pylori* among GERD patients and control group, *Cag A *and* Cag E* between the two groups, Fisher’s exact test and Chi-Square were used. A *P *value of <0.05 was considered as significant. 

## Results

**Figure 1:**
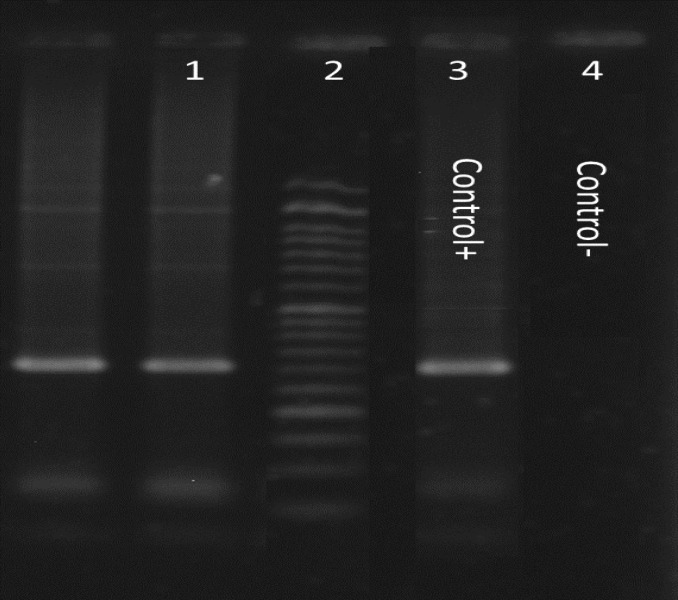
PCR *results* for the *ureC* gene in *H. pylori* isolates.

**Figure 2:**
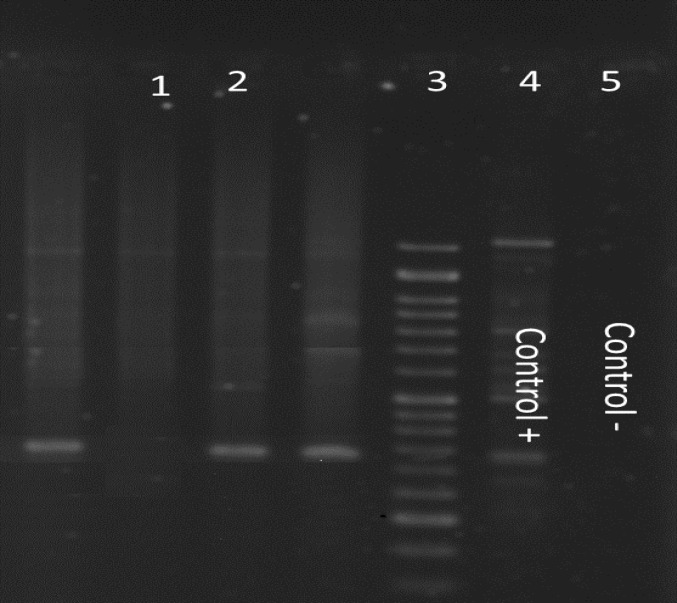
PCR results for the *cagA* gene in *H. pylori* isolates.

**Figure 3:**
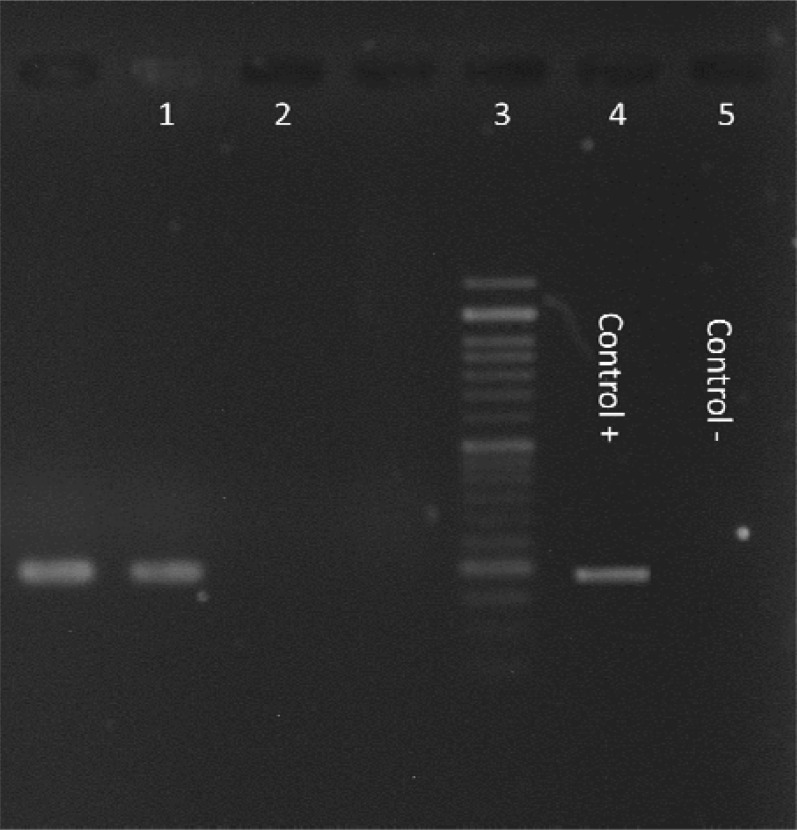
PCR results for the *cagE* gene in *H. pylori* isolates.

Out of 50 GERD patients, 42% were male (n=21) and 58% female (n=29) with the mean age of 45.78 ±22 years. In the control group, 54% were male and 46% female with mean age of patients with the mean age of 41.27± 18 years. The *ure C* PCR results in the GERD group showed that in 54% (n=27) of patient samples, *H. Pylori* DNA was detected whereas 62% (n=31) of the control group showed positive results for *ure C* gene (figure 1). And about *ure C* in control group and GERD group we cannot found statistical significance was seen in presence of *H. pylori* between the GERD and control groups. Prevalence of the c*ag A* gene among GERD patients was 44.4% (n=12) whereas among the control group was 87% (n=27) (figure 2). Prevalence of the c*ag E* among GERD patient was 44.4% (n=12) whereas among the control patients it was 64% (n=20) (figure 3). Coexistence of the c*ag A *and* cag* E as in GERD patients was 25.7% (n=7) and in the control patients it was 54.8% (n=17) (Table 2).

## Discussion

*H. pylori* infection plays a major role in the pathogenesis of peptic ulcer disease, chronic gastritis, and development of gastric cancer. However, its role in reflux diseases such as GERD is not clear (12-14). 

Line1: positive sample for the *ure C*, Line2: Size marker (50bp), Line 3: Positive cont*r*ol for *ure C*, Line4: Negative control for the *ure.*

GERD reduces the patients’ quality of life and imparts a significant economic burden on the healthcare system (15–17). Bacterial virulence and host inflammatory responses are important in determining the patterns of acid secretion and gastritis (18).

The incidence of *H. pylori* infection in patients with GERD varies widely from 30 to 90% (19). Geographical location of the studies due to the difference in the prevalence of *H. pylori* in the world is the reason of this heterogeneity (20). These epidemiological data do not support a causative role of *H. pylori* for reflux disease, but they suggest a negative association (21). Consistent to Johnson LF et al. study, we did not find a significant association between *H. pylori* prevalence among the GERD patients and control group. Other researchers have even found a lower incidence of *H. pylori* infection in patients with GERD and have suggested a ''protective'' role of *H. pylori* against the GERD (22، 23). Their findings are inconsistent with our results. 

In Asian populations, in contrast to Far East and European populations, patients with gastric ulcer get complicated by corpus-predominant gastritis, which is characterized by atrophy of acid-secreting glands due to gastric acid hypo-secretion (24،^25^). Gastric acid hypo-secretion prevents the development of GERD. According to previous Asian population-based studies, the prevalence of GERD is reported to have a lower prevalence (26،^29^); this confirms the theory.

Also, *H. pylori* and GERD have been found to be negatively associated and strongly dependent on cytotoxin-associated gene product *Cag* *A* positive strains (8). Recently, a study reported that *H. pylori* *Cag* *A* positive may potentially protect against development of GERD (30،^31^). According to previous studies, most *Cag A* positive strains in Asian countries were East Asian *Cag A* positive strains which can protect people against GERD (18). On the other hand, it has been reported that eradication of *Cag A* positive *H. pylori* strains is a risk factor for newly developed GERD (32،^33^). A meta-analysis study demonstrated that eradication of *Cag A* positive *H. pylori* was related to a higher risk of developing GERD in Asian studies (34). Also another study demonstrated a strong negative association between Barrett’s esophagus or erosive esophagitis and *H. pylori*, particularly in *Cag A* positive strains (35). our finding in Iranian subjects, similar to many studies as well as Xie T et al. report, showed that the cag* A* positive *H. pylori* strains were less common among GERD patients in comparison to the control group (*P *= 0.0001) but we did not find any association between *Cag E* in the GERD patients and control group. The co-existence of *Cag A* and *Cag E* in the control group was more than GERD patients significantly (*P* = 0.034). Our finding was supportive for the protective role of *H. pylori* with the *cagA /cagE* positive genotype against GERD development. Anita P et al showed prevalence of *cagE* in GERD patients is more from the genes studied, but no association was detected between *cagE* genotypes and clinical outcome (43). The paradox is that our study is probably due to the geographical distance between the two studies

 In Conclusion, we evaluated the prevalence of the *Cag A *and* Cag E* genes of *H. pylori* among GERD patients. We didn’t find any correlation between *H. pylori* frequency in GERD patients in comparison with the control group. However in accordance with several Asian studies, *H. pylori* strains from GERD patients were less positive for *cagA* gene as well as co-existence of *cagA/cagE* compared with the control group indicating probably protective role of these factor against GERD. However, more studies are needed to confirming this correlation and finding the possible mechanisms accurately.
